# A Linguistic Analysis Comparing Medical Students and Consultants in Explanations of Depression

**DOI:** 10.1192/bjo.2026.11114

**Published:** 2026-06-30

**Authors:** Alessandro Pace

**Affiliations:** University of Birmingham, Birmingham, United Kingdom

## Abstract

**Aims:**

Clinical depression is one of the most prevalent psychiatric illnesses, yet patient understanding of the condition is often lacking. Effectively educating patients on their diagnosis is a key communication skill for clinicians and future doctors.

This qualitative study aimed to compare how consultants and medical students, two ends of the medical hierarchy, differ when explaining depression to patients, whilst examining the potential clinical and educational implications.

**Methods:**

Semi-structured online interviews were conducted with 12 final-year students and four consultants (two psychiatrists and two general practitioners). Participants were asked to role-play how they would discuss a suspected depression diagnosis to a lay patient, and explain the condition.

Linguistic and thematic analysis was conducted using Nvivo.

**Results:**

Consultants tended to avoid heavy neurobiological discussion, using simplifications and generalisations to explain things with brevity and relating back to a patient’s individual experience. One psychiatrist explained that this simplified description aimed to prioritise patient comprehension and acceptance to treatment, where ‘*bogg[ing] them down with* *…* *scientific facts*’ was feared to cause confusion or take emphasis from what the clinician deemed important.

In contrast, despite avoiding medical jargon, students gave more verbose biochemical explanations. All students referred to the neurobiological theory behind depression within their answers.‘*Serotonin*’ specifically was mentioned by three students, but not by any consultants. Students also showed difference in language style, often asserting a more declarative tone by informing a patient of their diagnosis, clearly separating themselves as the expert. Consultants framed explanations as a collaboration and promoted patient self-reflection, inviting the patient to follow their diagnostic conclusion, reducing doctor–patient asymmetry in the hypothetical consultation.

**Conclusion:**

Students medicalised depression to properly inform patients and validate their experiences. Comparatively, consultants were succinct and practical for clarity, comprehension and mutual understanding. There is research to support this latter approach, which suggests that an overemphasis on the neurobiological basis of depression can cause a lack of focus on the social and emotional dimensions of the illness. This may even cause a favouring of pharmacological over cognitive therapies.

Communication skills training for these students has effectively taught them to avoid unexplained medical jargon, but may need greater emphasis on delivering practical, patient-centred information when educating patients on their psychiatric conditions. However, due to the limited scope of this study, further research would be needed with larger cohorts in a more clinically realistic setting.

